# A nomogram to predict arterial bleeding in patients with pelvic fractures after blunt trauma: a retrospective cohort study

**DOI:** 10.1186/s13018-021-02247-2

**Published:** 2021-02-08

**Authors:** Myoung Jun Kim, Jae Gil Lee, Eun Hwa Kim, Seung Hwan Lee

**Affiliations:** 1 Department of Surgery, Yonsei University Wonju College of Medicine, Wonju Severance Christian Hospital, Wonju, Republic of Korea; 2 Department of Surgery, Yonsei University College of Medicine, Seoul, Republic of Korea; 3 Biostatistics Collaboration Unit, Yonsei University College of Medicine, Seoul, Republic of Korea; 4 Department of Trauma Surgery, Gachon University Gil Medical Center, 21, Namdong-daero 774beon-gil, Namdong-gu, Incheon, Republic of Korea

**Keywords:** Pelvis, Fracture, Nomogram, Hemorrhage

## Abstract

**Background:**

Pelvic bone fractures are one of the biggest challenges faced by trauma surgeons. Especially, the presence of bleeding and hemodynamic instability features is associated with high morbidity and mortality in patients with pelvic fractures. However, prediction of the occurrence of arterial bleeding causing massive hemorrhage in patients with pelvic fractures is difficult. Therefore, the aim of this study was to develop a nomogram to predict arterial bleeding in patients with pelvic bone fractures after blunt trauma.

**Methods:**

The medical records of 1404 trauma patients treated between January 2013 and August 2017 were retrospectively reviewed. Patients older than 15 years with a pelvic fracture due to blunt trauma were enrolled (*n* = 148). The pelvic fracture pattern on anteroposterior radiography was classified according to the Orthopedic Trauma Association/Arbeitsgemeinschaft fur Osteosynthesefragen (OTA/AO) system. Multivariable logistic regression modeling was used to determine the independent risk factors for arterial bleeding. A nomogram was constructed based on the identified risk factors.

**Results:**

The most common pelvic fracture pattern was type A (58.8%), followed by types B (34.5%) and C (6.7%). Of the 148 patients, 28 (18.9%) showed pelvic arterial bleeding on contrast-enhanced computed tomography or angiography, or in the operative findings. The independent risk factors for arterial bleeding were a type B or C pelvic fracture pattern, body temperature < 36 °C, and serum lactate level > 3.4 mmol/L. A nomogram was developed using these three parameters, along with a systolic blood pressure < 90 mmHg. The area under the receiver operating characteristic curve of the predictive model for discrimination was 0.8579. The maximal Youden index was 0.1527, corresponding to a cutoff value of 68.65 points, which was considered the optimal cutoff value for predicting the occurrence of arterial bleeding in patients with pelvic bone fractures.

**Conclusions:**

The developed nomogram, which was based on the initial clinical findings identifying risk factors for arterial bleeding, is expected to be helpful in rapidly establishing a treatment plan and improving the prognosis for patients with pelvic bone fractures.

**Supplementary Information:**

The online version contains supplementary material available at 10.1186/s13018-021-02247-2.

## Background

Pelvic bone fractures can be a significant cause of bleeding and death and are currently among the biggest challenges faced by trauma surgeons [1, 2]. The mortality rate of patients with a pelvic fracture is 6–14%, and ranges from 54 to 70% in hemodynamically unstable patients with severe pelvic bone fractures [3–5]. Although in most cases bleeding from pelvic fractures is venous in origin, arterial bleeding is more common in patients with hemodynamic instability or ongoing hemorrhage despite pelvic binding. If pelvic arterial bleeding can be identified early, it can be rapidly treated using angiography and embolization [4]. Therefore, careful evaluation of the presence of bleeding in pelvic ring fractures, as well as its origin, is necessary for adequate management.

However, it is difficult to rapidly identify (or distinguish) the origin of bleeding (whether it is of venous, bone, or arterial origin) following a pelvic fracture. Although recent studies have reported on the predictive factors of arterial bleeding in trauma patients with pelvic bone fractures [3, 6–9], the usefulness of these predictors in a clinical setting remains unclear. Thus, there is a need for a simple and convenient scoring system to predict arterial bleeding in patients with pelvic bone fracture in the initial post-injury period.

Therefore, we investigated the associations between the initial clinical/laboratory findings and pelvic arterial bleeding in patients with pelvic bone fractures after blunt trauma. We hypothesized that a nomogram constructed with the identified risk factors would aid in the early prediction of arterial bleeding caused by a pelvic bone fracture.

## Methods

### Patient enrolment and data collection

Records of consecutive patients aged 15 years or older with traumatic injury who were treated between January 2013 and May 2017 at a single center in an urban setting in Seoul, South Korea, were retrospectively reviewed. This study was approved by the Institutional Review Board of Severance Hospital, Yonsei University Health System (4-2018-0114); the requirement for informed consent was waived because of the retrospective nature of the study.

**Fig. 1:**
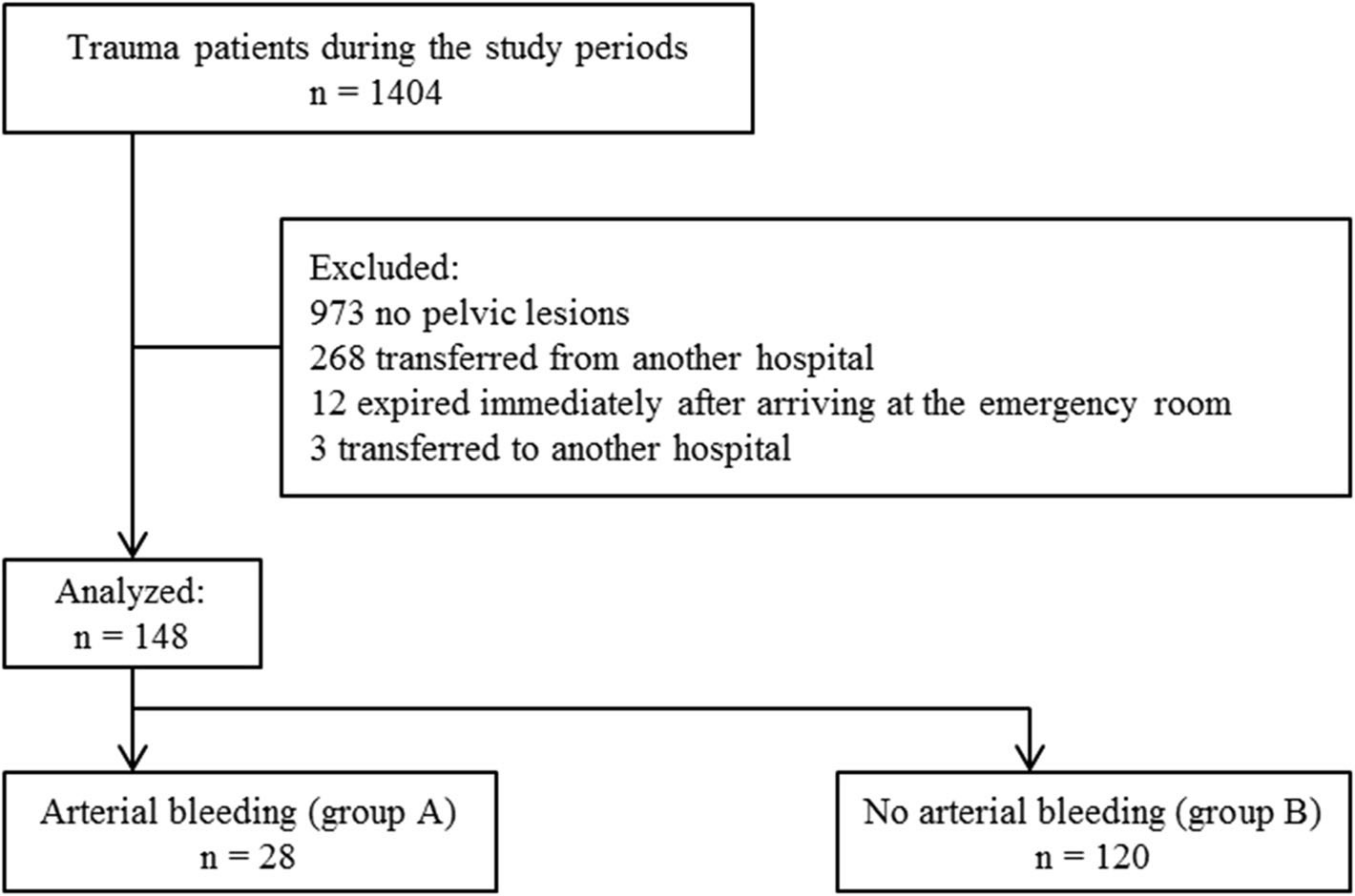
Patient selection flowchart

A total of 1404 trauma patients was admitted to the emergency department during the study period (Fig. 1). Primary and/or secondary surveys were conducted on these patients, according to the advanced trauma life support guidelines [10]. Of the 1404 patients, 268 patients who were managed or evaluated at other hospitals and 973 patients without lesions in the pelvic ring were excluded. Additionally, 3 patients who were referred to another hospital and 12 patients who died within minutes after arrival to the emergency room (ER) were excluded. Thus, 148 patients were enrolled. The patients were divided into groups A and B. Group A comprised of patients with pelvic arterial bleeding evident on contrast-enhanced computed tomography (CT) or angiography or in the operative findings. Group B comprised of patients without evidence of pelvic arterial bleeding.

### Clinical variables

Data collection included the following variables: sex, age, injury mechanism, initial vital signs, Glasgow Coma Scale (GCS) score, current medication, Abbreviated Injury Scale (AIS) score, Injury Severity Score (ISS), Revised Trauma Score (RTS), Trauma and Injury Severity Score (TRISS), Acute Physiology and Chronic Health Evaluation (APACHE) II score, and initial arterial blood values, including the serum lactate level. Additionally, using anteroposterior (AP) pelvic radiographs, the pelvic fracture pattern was classified as type A, B, or C, in accordance with the Orthopedic Trauma Association/Arbeitsgemeinschaft fur Osteosynthesefragen (OTA/AO) system [see Additional file 1]. The fracture pattern classification was on the basis of the findings of the trauma and orthopedic surgeons and was additionally confirmed by the surgical and radiological records.

### Statistical analysis and model construction

Continuous variables are expressed as means ± standard deviations or as medians and interquartile ranges (depending on the data distribution) and were evaluated using the Student *t* test or Mann-Whitney *U* test, as appropriate. Categorical variables are expressed as numbers (%) and were evaluated using the chi-square test or Fisher’s exact test, as appropriate. Univariable logistic regression analyses were performed to identify the factors associated with the risk of arterial bleeding. A multivariable logistic regression analysis was conducted using variables with clinical meaning and/or statistical significance in the univariable analyses. A nomogram was constructed based on the results of the multivariable logistic regression analysis. The nomogram was internally validated for discrimination and calibration via bootstrapping (500 resamples) [11]. The area under the receiver operating characteristic (ROC) curve (AUC) was used to assess the predictive accuracy of the model. The relationship between the observed probability of arterial bleeding and the predicted probability was graphically assessed using a calibration plot. To improve the clinical practicality of the nomogram, we assigned cutoff values for continuous variables, derived from the Youden index [12]. The accuracy of the optimal cutoff value was assessed by the sensitivity, specificity, predictive values, and likelihood ratios. All statistical tests were 2-sided, and *P* values less than 0.05 were considered statistically significant. The analyses were performed using SAS version 9.4 (SAS Institute Inc., Cary, NC, USA) and the R version 3.4 software (http://www.r-project.org/) with the “rms” package, which was used to construct the nomogram.

## Results

### Baseline characteristics

The baseline characteristics of the patients are shown in Table 1. Among the 148 patients, 28 patients (18.9%) had arterial bleeding (group A) and 120 patients (81.1%) did not have arterial bleeding (group B). The most common mechanism of injury was being struck by a motor vehicle as a pedestrian (39.9%), followed by falls (35.8%), motorcycle crashes (15.5%), motor vehicle crashes (6.1%), and other mechanisms (2.7%). The overall in-hospital mortality rate was 5.4%, and the in-hospital mortality rate was significantly higher in group A than in group B (32.1% vs. 7.5%, *P* = 0.001).

**Table 1:** Baseline characteristics of patients

Variable	Group A (*n* = 28) (arterial bleeding)	Group B (*n* = 120) (no arterial bleeding)	*P* value
Age (years)	57.5 (30.5, 74.0)	49.5 (33.0, 65.0)	0.426
Sex			0.342
Male	16 (57.1)	80 (66.7)	
Female	12 (42.9)	40 (33.3)	
Medications			
Aspirin	2 (7.1)	3 (2.5)	0.239
Clopidogrel	1 (3.6)	2 (1.7)	0.469
Warfarin	0 (0)	2 (1.7)	> 0.999
Injury mechanism			0.191
Pedestrian struck by a motor vehicle	14 (50.0)	45 (37.5)	
Motor vehicle crashes	0 (0)	9 (7.5)	
Motorcycle crashes	3 (10.7)	20 (16.7)	
Falls	9 (32.1)	44 (36.7)	
Others	2 (7.1)	2 (1.7)	
AIS			
Head and neck	2 (0, 2.0)	1 (0, 2.0)	0.619
Face	0 (0, 0)	0 (0, 1.0)	0.008
Chest	2 (0, 3.0)	0 (0, 3.0)	0.408
Abdomen	3 (0, 3.0)	0 (0, 2.0)	0.001
Extremities	4 (2.5, 4.0)	2 (2.0, 3.0)	< 0.001
External	1 (1.0, 1.0)	1 (0.5, 1.0)	0.213
GCS score	15.0 (12.0, 15.0)	15.0 (14.0, 15.0)	0.203
ISS	28 (18.0, 34.0)	15.5 (9.0, 25.0)	0.001
RTS	6.64 (5.44, 7.84)	7.84 (7.11, 7.84)	0.006
TRISS	88.2 (57.09, 95.94)	96.75 (89.0, 99.25)	0.001
APACHE II	23.0 ± 12.9	15.7 ± 8.2	0.005
In-hospital mortality	9 (32.1)	9 (7.5)	0.001

Values are presented as mean ± SD, median (interquartile range), or *n* (%)

*AIS* Abbreviated Injury Scale, *ISS* Injury Severity Score, *RTS* Revised Trauma Score, *TRISS* Trauma and Injury Severity Score, *APACHE Acute* Physiology and Chronic Health Evaluation, *GCS* Glasgow Coma Scale

### Initial clinical and laboratory variables

Initial clinical and laboratory variables were compared between the two groups (Table 2). Significant group differences were found for the following variables: a systolic blood pressure (SBP) < 90 mmHg, heart rate (HR) > 120 bpm, body temperature (BT) < 36 °C, serum lactate level > 3.4 mmol/L, and base deficit < − 6 mmol/L. According to the classification of the posterior pelvic ring stability using the OTA/AO system, the most common fracture pattern in group A was type B, with partial instability (*n* = 14, 50.0%), followed by types A (*n* = 7, 25.0%) and C (*n* = 7, 25.0%). Type A (*n* = 80, 66.7%) was the most common fracture pattern in group B, followed by types B (*n* = 37, 30.8%) and C (*n* = 3, 2.5%), with a significant group difference in the fracture pattern distribution (*P* < 0.001).

**Table 2:** Initial clinical and laboratory findings

Variable	Group A (*n* = 28) (arterial bleeding)	Group B (*n* = 120) (no arterial bleeding)	*P* value
SBP < 90 mmHg	13 (46.4)	22 (18.3)	0.002
Heart rate > 120 bpm	4 (14.3)	11 (9.2)	0.419
BT < 36 °C	14 (50.0)	19 (15.8)	< 0.001
GCS score	15.00 (14.00, 15.00)	15.00 (12.00, 15.00)	0.203
Lactate > 3.4 mmol/L	17 (60.7)	32 (26.7)	0.001
Base deficit < − 6 mmol/L	10 (35.7)	15 (12.5)	0.003
Pelvic fracture pattern			< 0.001
A	7 (25.0)	80 (66.7)	
B	14 (50.0)	37 (30.8)	
C	7 (25.0)	3 (2.5)	

Values are presented median (interquartile range) or *n* (%)

*SBP* systolic blood pressure, *BT* body temperature, *GCS* Glasgow coma scale

### Univariable and multivariable logistic regression analyses for the risk factors for pelvic arterial bleeding

The results of the univariable and multivariable logistic regression analyses are presented in Table 3. The multivariable analysis revealed type B (odds ratio [OR] = 3.404, 95% confidence interval [CI] = 1.116–10.384, *P* = 0.031) and type C fracture patterns (OR = 22.534, 95% CI = 3.606–140.804, *P* = 0.001) as independent risk factors for pelvic arterial bleeding. Among the initial clinical and laboratory variables, BT < 36 °C (OR = 8.757, 95% CI = 2.718–28.216, *P* < 0.001) and a serum lactate > 3.4 mmol/L (OR = 4.589, 95% CI = 1.354–15.552, *P* = 0.014) were revealed as independent risk factors for pelvic arterial bleeding.

**Table 3:** Univariable and multivariable logistic regression analyses of the risk factors for pelvic arterial bleeding

Variable	Univariable analysis		Multivariable analysis	
	OR (95% CI)	*P* value	OR (95% CI)	*P* value
SBP < 90 mmHg	3.861 (1.610–9.260)	0.002	1.338 (0.352–5.078)	0.669
Heart rate > 120 bpm	1.652 (0.484–5.632)	0.423		
BT < 36 °C	5.316 (2.187–12.922)	< 0.001	8.757 (2.718–28.216)	< 0.001
GCS score	0.964 (0.876–1.062)	0.463		
Lactate > 3.4 mmol/L	4.250 (1.799–10.039)	0.001	4.589 (1.354–15.552)	0.014
Base deficit < − 6 mmol/L	3.889 (1.514–9.991)	0.005	1.263 (0.303–5.259)	0.749
Pelvic fracture pattern				
A	Ref.			
B	4.324 (1.611–11.608)	0.004	3.404 (1.116–10.384)	0.031
C	26.667 (5.617–126.596)	< 0.001	22.534 (3.606–140.804)	0.001

*OR* odds ratio, *CI* confidence interval, *SBP* systolic blood pressure, *BPM* beats per minute, *BT* body temperature, *GCS* Glasgow coma scale

### Development and validation of a nomogram for predicting pelvic arterial bleeding

**Fig. 2:**
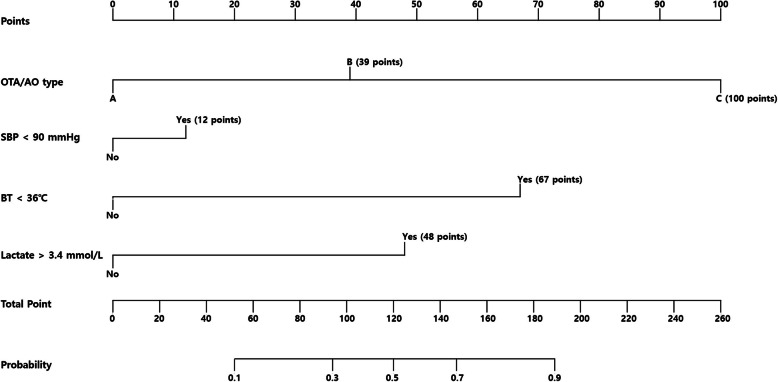
Nomogram to predict arterial bleeding in patients with pelvic bone fractures. *SBP* systolic blood pressure, *BT* body temperature, *OTA/AO* Orthopedic Trauma Association/Arbeitsgemeinschaft für Osteosynthesefragen

**Fig. 3:**
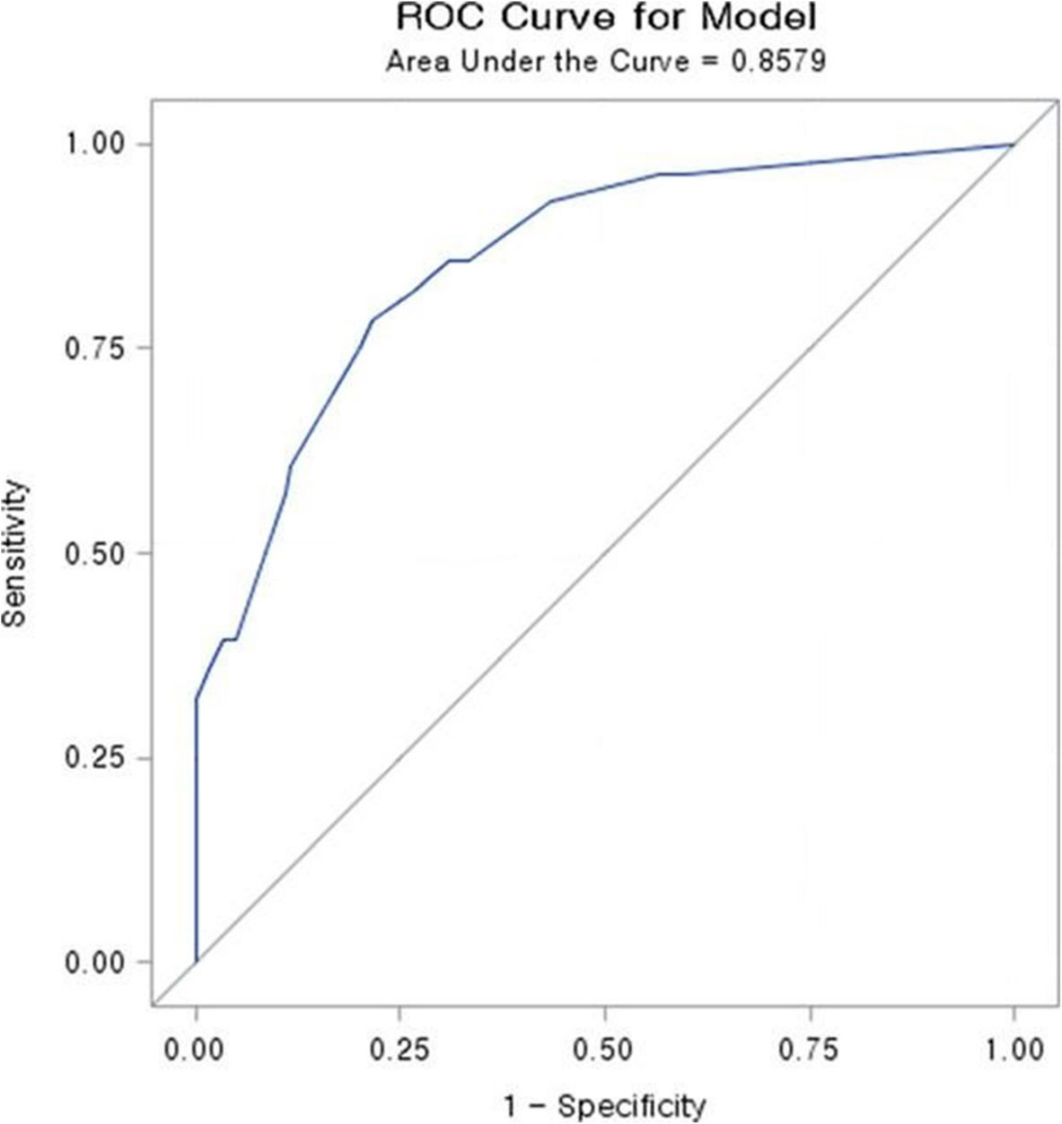
Receiver operating characteristic (ROC) curves for the predictive model

**Fig. 4:**
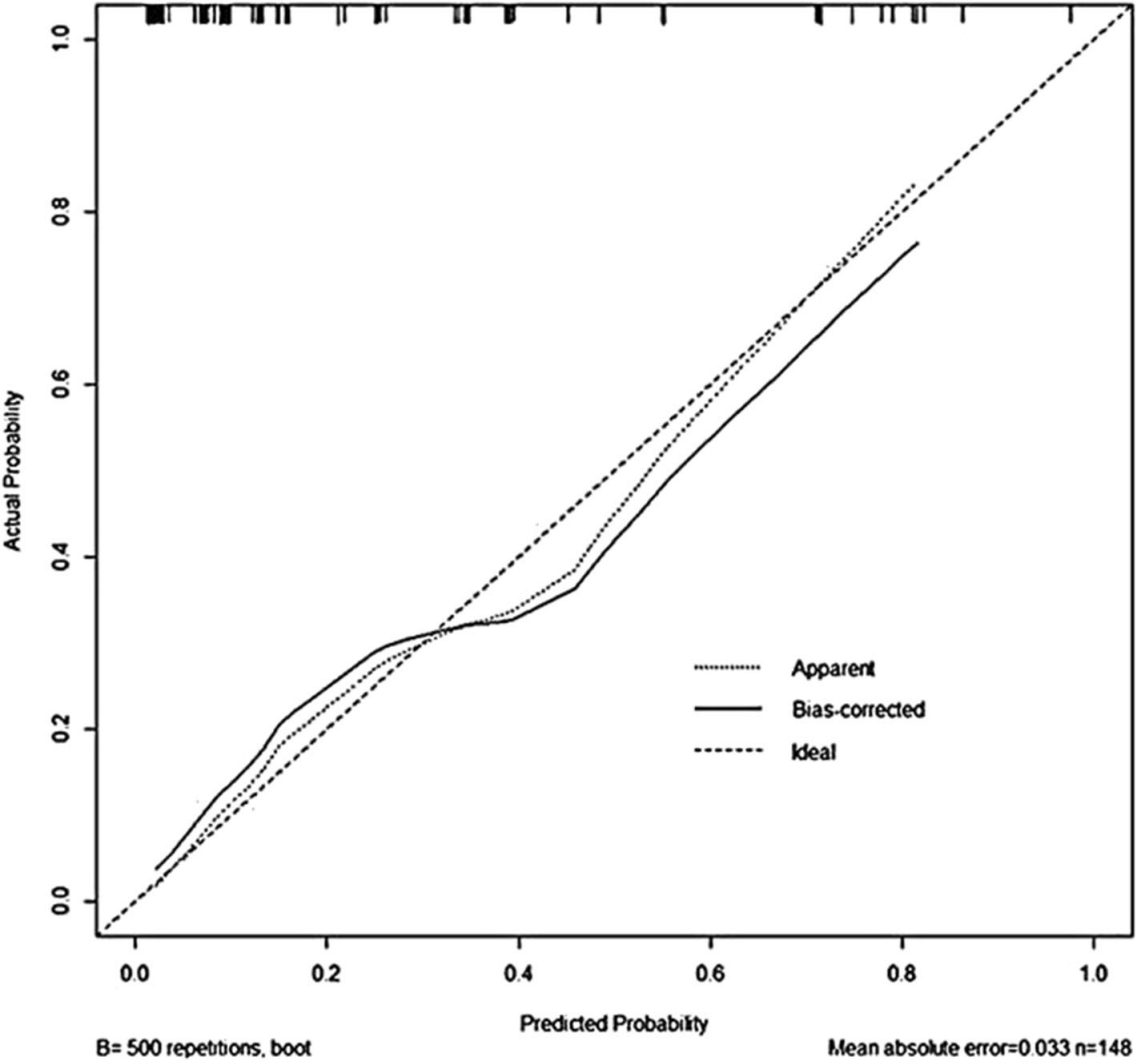
Calibration plot in the internal validation

A nomogram to predict pelvic arterial bleeding was developed using the identified independent risk factors for pelvic arterial bleeding (pelvic bone fracture pattern, BT, and serum lactate level) and SBP, as this parameter is considered clinically important (Fig. 2). The overall predictive accuracy of the model incorporating these four variables was good, with an AUC of 0.8579 (95% CI = 0.7814–0.9343) (Fig. 3). Additionally, the calibration plot in the internal validation demonstrated a good correlation between the observed probability of arterial bleeding and the predicted probability, as assessed in a bootstrap-corrected ROC curve (AUC = 0.8712, 95% CI = 0.7880–0.9373) (Fig. 4).

### Risk of pelvic arterial bleeding based on the nomogram score

The optimal cutoff value of the total nomogram score was 68.65, which had a maximal Youden index of 0.1527. Using a cutoff of > 68.65, the nomogram had a sensitivity of 78.6%, specificity of 78.3%, positive predictive value of 45.8%, and negative predictive value of 94.0% (Table 4).

**Table 4:** Accuracy of the nomogram score in the estimation of the risk of pelvic arterial bleeding

Variable	Value (95% CI)
Area under ROC curve, concordance index	0.8579 (0.7814–0.9343)
Cutoff score	68.65
Sensitivity, %	78.6 (60.5–89.8)
Specificity, %	78.3 (70.1–84.8)
Positive predictive value, %	45.8 (32.6–59.7)
Negative predictive value, %	94.0 (85.5–97.2)
Positive likelihood ratio	3.622 (2.452–5.363)
Negative likelihood ratio	0.273 (0.134–0.559)

*ROC* receiver operating curve, *CI* confidence interval

## Discussion

In the past decade, several studies have investigated the risk factors predicting pelvic hemorrhage following pelvic injury [6–9, 13–19]; however, controversy remains regarding whether each risk factor is sufficient and adequately convenient to allow the rapid identification of the origin of pelvic bleeding in the acute trauma setting. A rapid and accurate diagnosis and management of pelvic bleeding is critical for the optimization of patient survival [20–22]. Unfortunately, the early identification of hemorrhage in patients with pelvic fractures is often difficult. Delays in the recognition and treatment of pelvic bleeding result in the progression from compensated reversible shock to multiple organ failure, and eventually, death. Particularly, unstable pelvic fractures caused by a high-energy injury may lead to severe bleeding and irreversible hemorrhagic shock [23, 24].

In the present study, we investigated the associations between pelvic arterial bleeding and the initial laboratory and clinical findings, including the pelvic bone fracture pattern on AP pelvic radiography, performed during an initial visit to the ER after trauma. A pelvic fracture pattern of type B or type C, BT < 36 °C, and serum lactate level > 3.4 mmol/L were identified as independent risk factors for pelvic arterial bleeding after pelvic trauma. Moreover, we developed a nomogram to predict early pelvic arterial bleeding, based on the three identified independent risk factors and SBP < 90 mmHg. The nomogram demonstrated good accuracy in estimating the risk of pelvic arterial bleeding. In addition, the nomogram was internally validated and showed good performance in terms of calibration and discrimination.

Previous studies have shown that unstable pelvic fractures are associated with hemodynamic instability [6, 14–19]. Unstable pelvic fractures are known to be accompanied by major ligament disruptions and are significantly associated with the occurrence of pelvic fracture-related arterial bleeding and the need for pelvic embolization [7, 17–19]. Furthermore, a previous study reported that a pelvic fracture pattern with major ligament disruption, such as the sacroiliac ligament, is an independent risk factor for pelvic arterial bleeding [3]. In the OTA/AO classification system, type B and type C fractures, as unstable pelvic fractures, are more likely to be associated with vascular injuries than are type A fractures. Consistent with this, the present study revealed type B and type C fractures (according to the OTA/AO system) as independent risk factors for pelvic arterial bleeding in patients with blunt pelvic trauma. We used OTA/AO classification rather than Young-Burgess classification, as the OTA/OA system is easier use with pelvic AP radiographs and better reflects the hemodynamic status [14–16].

Hypothermia inhibits coagulation protease activity and platelet function [25, 26]. Mortality from traumatic hemorrhage is markedly increased in severe hypothermia when core temperatures fall below 32 °C [27]. Furthermore, clinically significant effects on plasma coagulation, platelet function, and clinical bleeding are seen in moderate hypothermia at temperatures below 34 °C [25–32]. However, a BT of less than 36 °C was significantly associated with pelvic arterial bleeding after pelvic ring fractures in the present study. Although a BT < 36 °C had the highest points in the nomogram, hypothermia alone did not meet the scoring criteria for the prediction of pelvic arterial bleeding. Similarly, the other parameters in the nomogram, with the exception of a type C fracture, failed to meet the scoring criteria when considered alone. Therefore, it is necessary to simultaneously consider various risk factors to improve the prediction of pelvic arterial bleeding.

Recent clinical guidelines recommend measuring the serum lactate level or base deficit as a sensitive test to monitor the degree of bleeding and shock [22, 33, 34]. The amount of lactic acid produced by anaerobic decomposition is an indirect indicator of oxygen debt, tissue low perfusion, and hemorrhagic shock. Furthermore, previous studies have demonstrated that an increased serum lactate level is associated with pelvic bleeding caused by pelvic trauma [8, 35]. Consistent with this, the present study revealed a serum lactate level > 3.4 mmol/L as an independent risk factor of arterial bleeding in blunt trauma patients with pelvic bone fractures.

In general, it is accepted that trauma patients with SBP < 90 mmHg are in hypotension. Recent studies have suggested that initial SBP in the range of 90–110 mmHg, or less, in a trauma patient may be indicative of hypoperfusion and is associated with poor patient outcomes [3, 9, 13, 36, 37]. Although different SBP cutoff values were utilized, previous studies have reported that decreased SBP is an independent risk factor of arterial bleeding in patients with pelvic bone fractures [3, 9, 13]. In the present study, univariate analysis revealed that patients with and without arterial bleeding had significant differences in the frequency of SBP < 90 mmHg. However, SBP < 90 mmHg was not identified as a significant independent risk factor in multivariable analysis. Despite this, we included SBP < 90 mmHg in the parameters of the nomogram, given its proven clinical significance [9, 36, 37], which resulted in the unabated good predictability of the nomogram.

To the best of our knowledge, no study has assessed the risk of arterial bleeding in patients with pelvic bone fractures using a nomogram. This nomogram can be easily and quickly used to predict the occurrence of arterial bleeding in the acute management stage in patients with pelvic bone fractures. Based on the present study data, we suggest that a nomogram score of greater than 68.65 points during the initial assessment of patients with pelvic bone fractures after blunt trauma necessitates the preparation for the control of pelvic arterial bleeding. However, there are some important issues to consider before using the nomogram. The clinical use of the model requires pelvic fractures to be diagnosed as a type A, B, or C fracture via AP pelvic radiographs. Additionally, although pelvic arterial bleeding can occur in all pelvic fracture patterns and occurred in 25% of patients with type A fracture in group A, “fracture type A” was used as the reference, given its stability and hemodynamic status. Furthermore, other sources of bleeding (beyond pelvic bleeding) should be considered as another potential cause of hemorrhagic shock in patients with multiple traumas. Furthermore, at least 1 in 10 patients with pelvic bone fractures and arterial bleeding may be potentially missed (sensitivity, 0.786; negative predictive value, 0.94) with the rigid application of the nomogram. Therefore, the nomogram results should be carefully interpreted and used strictly in wider context of the patient’s clinical condition, clinical setting, individual-included factors, and factors not included in the nomogram.

The present study has some limitations. First, the present study was retrospective in nature. Second, the present study analysis was based on data from a single institution, and the study population was relatively small. Therefore, it may be difficult to generalize the study results. Third, our calibration plot was only validated internally. Thus, we are planning a prospective study to validate the model externally.

## Conclusions

We developed a nomogram to predict the risk of arterial bleeding in patients with pelvic bone fractures. This nomogram consists of reliable parameters, and it can aid physicians by allowing easier and more rapid decision-making in the management of patients with pelvic bone fractures after blunt trauma. However, this nomogram should be validated in a prospective, multicenter randomized study.

## Supplementary Information

**Additional file 1. **OTA/AO classification of pelvic fracture. *OTA/AO* Orthopedic Trauma Association/Arbeitsgemeinschaft für Osteosynthesefragen

## Data Availability

The datasets used and/or analyzed during the current study are available from the corresponding author on reasonable request.

## Abbreviations

AIS: Abbreviated Injury Scale
APACHE: Acute Physiology and Chronic Health Evaluation
AUC: Area under the receiver operating characteristic curve
BT: Body temperature
CI: Confidence interval
CT: Computed tomography
ER: Emergency room
GCS: Glasgow Coma Scale
HR: Heart rate
ISS: Injury Severity Score
OR: Odds ratio
OTA/AO: Orthopedic Trauma Association/Arbeitsgemeinschaft für Osteosynthesefragen
ROC: Receiver operating curve
RTS: Revised Trauma Score
SBP: Systolic blood pressure
TRISS: Trauma and Injury Severity Score
