# Supplementary material for: A nomogram to predict arterial bleeding in patients with pelvic fractures after blunt trauma: a retrospective cohort study
Source: J Orthop Surg Res. 2021 Feb 8;16:122. doi: 10.1186/s13018-021-02247-2 (PMC7869209; doi:10.1186/s13018-021-02247-2)
Supplement: Supplementary file 1 — Additional file 1. OTA/AO classification of pelvic fracture. OTA/AO Orthopedic Trauma Association/Arbeitsgemeinschaft für Osteosynthesefragen [file 13018_2021_2247_MOESM1_ESM.docx]

Additional file 1. OTA/AO classification of pelvic fracture

| **Type** | **Description** |
| --- | --- |
| Type A: stable - posterior arch is intact | A1: fracture does not involve the pelvic ring (avulsion fracture or fracture of the iliac wing) - A1.1: iliac spine - A1.2: iliac crest - A1.3: ischial tuberosity |
|  | A2: stable or minimally displaced fracture of the pelvic ring - A2.1: iliac wing fractures - A2.2: unilateral fracture of anterior arch - A2.3: bifocal fracture of anterior arch |
|  | A3: transverse fracture of the sacrum - A3.1: sacrococcygeal dislocation - A3.2: sacrum undisplaced - A3.3: sacrum displaced |
| Type B: rotationally unstable, vertically stable - incomplete disruption of the posterior arch | B1: open book injury (external rotation) - B1.1: sacroiliac joint, anterior disruption - B1.2: sacral fracture |
|  | B2: lateral compression injury (internal rotation) - B2.1: anterior compression fracture, sacrum - B2.2: partial sacroiliac joint fracture, subluxation - B2.3: incomplete posterior iliac fracture |
|  | B3: bilateral type B fracture - B3.1: bilateral open book fracture - B3.2: open book fracture and lateral compression - B3.3: bilateral lateral compression |
| Type C: rotationally and vertically unstable - complete disruption of the posterior arch | C1: unilateral fracture - C1.1: fracture of the iliac bone - C1.2: sacroiliac dislocation and/or fracture dislocation - C1.3: sacral fracture |
|  | C2: bilateral fracture with one side type B fracture (rotationally unstable) and one side type C fracture (vertically unstable) |
|  | C3: bilateral fracture with both sides type C fracture (both sides completely unstable) |

OTA/AO = Orthopedic Trauma Association/Arbeitsgemeinschaft für Osteosynthesefragen
